# Progression of Dementia Assessed by Temporal Correlations of Physical Activity: Results From a 3.5-Year, Longitudinal Randomized Controlled Trial

**DOI:** 10.1038/srep27742

**Published:** 2016-06-13

**Authors:** Kun Hu, Rixt F. Riemersma - van der Lek, Melissa Patxot, Peng Li, Steven A. Shea, Frank A. J. L. Scheer, Eus J. W. Van Someren

**Affiliations:** 1 Division of Sleep and Circadian Disorders, Brigham and Women’s Hospital; and Division of Sleep Medicine, Harvard Medical School, Boston, MA 02215, United States.; 2 Netherlands Institute for Neuroscience, Amsterdam, The Netherlands; 3 University of Groningen, University Medical Center Groningen, Department of Psychiatry, Groningen, The Netherlands; 4 Oregon Institute of Occupational Health Sciences, Oregon Health & Science University, Portland, OR 97239, United States; 5 Depts. of Integrative Neurophysiology and Psychiatry GGZ inGeest, Center for Neurogenomics and Cognitive Research (CNCR), Neuroscience Campus Amsterdam, VU University and Medical Center, Amsterdam, The Netherlands

## Abstract

Cross-sectional studies show that activity fluctuations in healthy young adults possess robust temporal correlations that become altered with aging, and in dementia and depression. This study was designed to test whether or not within-subject changes of activity correlations (i) track the clinical progression of dementia, (ii) reflect the alterations of depression symptoms in patients with dementia, and (iii) can be manipulated by clinical interventions aimed at stabilizing circadian rhythmicity and improving sleep in dementia, namely timed bright light therapy and melatonin supplementation. We examined 144 patients with dementia (70–96 years old) who were assigned to daily treatment with bright light, bedtime melatonin, both or placebos only in a 3.5-year double-blinded randomized clinical trial. We found that activity correlations at temporal scales <~2 hours significantly decreased over time and that light treatment attenuated the decrease by ~73%. Moreover, the decrease of temporal activity correlations positively correlated with the degrees of cognitive decline and worsening of mood though the associations were relatively weak. These results suggest a mechanistic link between multiscale activity regulation and circadian/sleep function in dementia patients. Whether temporal activity patterns allow unobtrusive, long-term monitoring of dementia progression and mood changes is worth further investigation.

Dementia and Alzheimer’s disease (AD) have become increasingly prevalent as the population aged 65 and older continues to increase^1^,^2^. To better monitor the onset and progression of dementia, regular assessments of cognitive function and behavior are encouraged, and tools for ambulatory monitoring while not interrupting daily life are desirable^3^. Here we examined whether a nonlinear measure of activity patterns based on temporal correlations in spontaneous motor activity fluctuations can be used to objectively assess cognitive and behavioral changes in patients with dementia.

Human motor activity displays seemingly irregular fluctuations over a wide range of time scales from seconds to hours^4^,^5^. In healthy young individuals these fluctuations are not random but possess robust temporal correlations that are similar at different time scales and independent of mean activity levels^6^,^7^. Mounting evidence suggests that altered temporal activity fluctuations reflect the changes in brain function that occur with aging and in diseases^5^,^7^,^8^,^9^,^10^,^11^,^12^,^13^,^14^. For instance, activity fluctuations in older individuals with dementia are more random with reduced correlations at time scales >~2 hours; and the perturbation is more pronounced in AD patients with higher cortical plaque density^9^,^10^. Recent studies also suggest clinical relevance of activity fluctuations at smaller time scales, showing altered activity patterns at <~2 hours in patients with mood disorders^5^,^11^,^12^,^13^,^14^. However, these findings are exclusively based on cross-sectional studies. The clinical relevance of temporal activity regulation in dementia awaits evidence from longitudinal studies that examine within-subject changes during the progression of dementia and the potential association with mood changes.

One possible common pathological pathway for the observed altered multiscale activity regulation in dementia and depression is disrupted function of the sleep/circadian control system^15^. There are several lines of studies supporting this hypothesis. (1) Cumulative evidence suggests a mechanistic role of sleep/circadian control in the development and progression of dementia^16^. For instance, disturbances in sleep and circadian rhythms are common in older persons and more pronounced in patients with dementia^17^,^18^,^19^. In addition, recent studies have shown that disturbed sleep and daily activity patterns may be an earliest sign of dementia or AD development^20^,^21^,^22^. (2) Circadian/sleep dysfunction is also associated with mood disturbances. Patients with depression often have abnormal sleep-wake cycles and reduced circadian amplitudes in daily activity and other physiological functions^17^,^18^,^19^,^23^,^24^,^25^; and treatment of depression with antidepressants helps to restore disrupted sleep and daily/circadian rhythms^18^,^19^. (3) Timed bright light therapy that can improve sleep and SCN functioning is also beneficial for mood and cognition^26^,^27^. (4) Most related to multiscale activity regulation, functional changes in the circadian pacemaker (suprachiasmatic nucleus; SCN) are accompanied by not only perturbed activity rhythms at ~24 hours^28^,^29^ but also degraded temporal activity correlations at multiple time scales^9^,^10^,^30^. Despite the evidence, it is unknown whether degraded activity regulation in dementia patients can be attenuated/reversed by manipulation of sleep/circadian regulation.

To better understand the clinical relevance of multiscale activity regulation and the underlying mechanisms, we examined data collected longitudinally to test four hypotheses: (1) temporal activity correlations decrease gradually over time in dementia patients; (2) long-term bright light with melatonin therapy attenuates the degradation in temporal activity correlations over time; (3) the degree of the decline in activity correlations positively correlates with the worsening of depression symptoms in patients/individuals with dementia; and (4) the degree of the decline in activity correlations positively correlates with cognitive declines. To test these hypotheses, we examined motor activity regulation of 165 patients (70–96 years old) with mid- to late-stage dementia from an existing database for a double-blind randomized clinical trial (controlled-trials.com/isrctn Identifier: ISRCTN93133646; Registered on December 09, 2005)^31^. These patients were assessed at baseline and every 6 months thereafter for up to 3.5 years. In this clinical trial, 45 patients received daily treatment with bright light, 39 took bedtime melatonin each day, 44 received both the bright-light and melatonin treatments, and 37 received placebos only. To estimate temporal correlations in the activity fluctuations at time scales from ~0.1 up to 12 hours, we performed the detrended fluctuation analysis (DFA) that has been widely used in physiological data analysis^15^. We determined the within-subject changes in multiscale activity correlations over time and related the results to the changes in mood and cognition of the same subjects.

## Results

### Temporal Correlations of Activity Fluctuations in Dementia at Baseline

**Figure 1:**
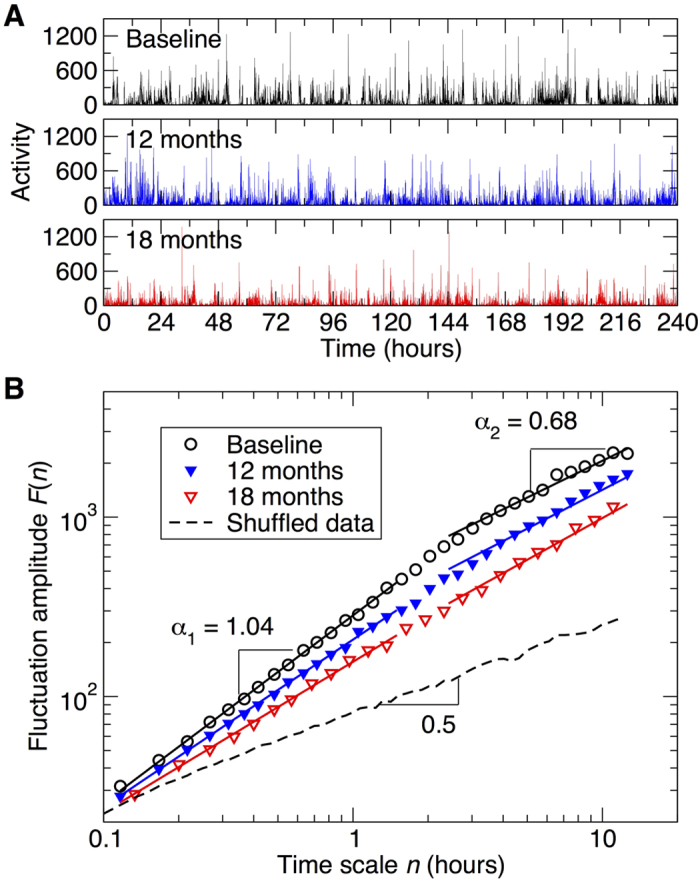
Changes in temporal activity correlations over time. (**A**) Activity recordings of a patient with Alzheimer’s disease in the group without light or melatonin treatments during baseline, 12-month, and 18-month assessments (**B**) DFA results of signals in (**A**). On the abscissa, n represents the time scale in hours. The fluctuation functions F(n) are vertically shifted for better visualization of changes in the slopes (DFA exponents) over time. The fluctuation function was also obtained from surrogate data that were generated from the baseline recording in A by randomly shuffling the data points. Shuffling destroyed the temporal organization of activity fluctuations, leading to white noise without correlations as indicated by a power-law function (i.e., a straight line in the log-log plot) with the slope of 0.5 (dashed line) over the entire range of tested time scales.

**Figure 2:**
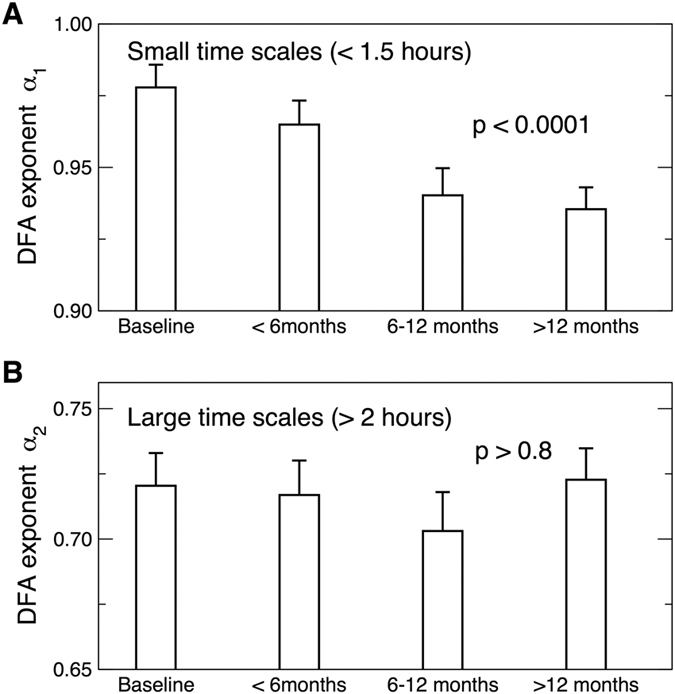
Correlations in activity fluctuations at baseline and during the follow-up assessments. (**A**) Correlations at time scales <2 hours, as characterized by the DFA exponent α1, decreased gradually over time. (**B**) Correlations at time scales >2 hours, as characterized by the DFA exponent α2, remained virtually unchanged over the period of the study. For better visualization, data during the follow-up assessments were divided into three bins (<6 months, 6–12 months, and >12 months). P values for the influences of time on the exponents were obtained using mixed models in which time into the study was included as a continuous variable (i.e., not binned categorized variable). Mean values were obtained from all individuals in all groups and error bars indicate standard errors (between individuals).

At baseline (i.e., prior to any intervention) activity fluctuations in these dementia patients had positive correlations at all tested time scales (as indicated by DFA exponent >0.5) but the temporal correlations were much weaker (i.e., more random fluctuations) at time scales >2 hours as compared to that at smaller time scales (DFA exponents: α_2_ = 0.72 ± 0.01 [SE] at >2 hours; α_1_ = 0.97 ± 0.01 at < 1.5 hours; α_1_ − α_2 = _0.25 ± 0.02; Paired t-test, p < 0.0001) (Figs 1 and 2). At baseline, α_1_ and α_2_ showed no significant differences between the four groups (both p > 0.7).

### Effect of Bright Light Therapy on Progressive Reduction of Activity Correlations

Temporal correlations of activity fluctuations at time scales <1.5 hours gradually decreased over the ~3.5 years of follow-up (Fig. 1 and 2) with a mean annual reduction of 0.021 ± 0.004 (SE) in α_1_ (Mixed Model; Time: t ratio = −4.61, p < 0.0001; Fig. 2A). In contrast, activity correlations at time scales >2 hours (α_2_) showed no significant change over the period of the study (p > 0.8; Fig. 2B).

**Figure 3:**
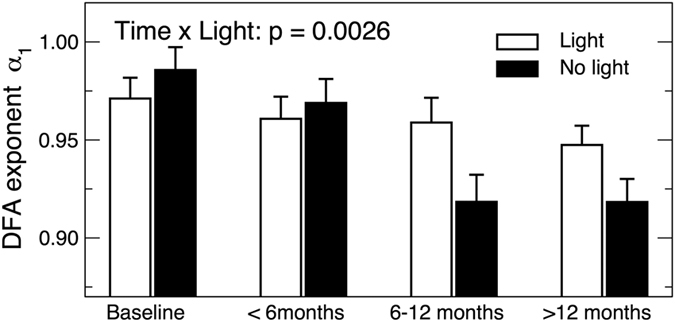
Effects of light treatment on activity correlations. P value is for the effect of the interaction between light and time course on DFA exponents. Data during the follow-up assessments were divided into three bins for better visualization (<6 months, 6–12 months, and >12 months). Error bars indicate standard errors between individuals.

The mixed model revealed an interaction between the effects of time and bright light treatment on α_1_ (Time × Light: 0.024 ± 0.008, 95% CI: 0.0083–0.039, t ratio = −3.02, p = 0.0026), i.e., α_1_ decreased by 0.033 ± 0.007 per year in the people who received the placebo light treatment and only by about a third, 0.009 ± 0.004 per year in the people who received the active bright light treatment (Fig. 3). These results indicate that bright light treatment counteracted the degradation of short-term activity correlations in dementia by 73% (95% CI: 25–118%).

Melatonin treatment did not affect the rate of change in α_1_ during follow-up (Melatonin × Time: p > 0.9) and did not affect the interaction between the effects of time and light treatment (Melatonin × Light × Time: p > 0.15). There were no significant effects of light, melatonin, and their interaction on the change of α_2_ (p values > 0.1 for Light × Time, Melatonin × Time, Melatonin × Light × Time).

### Associations Between Reduction in Activity Correlations and Mood and Cognitive Declines

**Figure 4:**
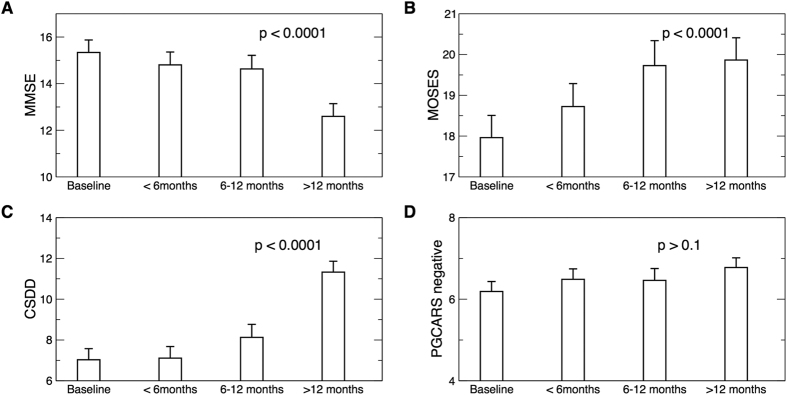
Functional outcome measures at baseline and during the follow-up assessments. (**A**) Mini-mental state examination (MMSE). (**B**) A subscale of the multidimensional observation scale for elderly subjects (MOSES) for withdrawn behavior. (**C**) Cornell Scale for Depression in Dementia (CSDD). (**D**) Negative affect scale of the Philadelphia Geriatric Centre Affect Rating Scale (PGCARS). Data during the follow-up assessments were divided into three bins for better visualization (<6 months, 6–12 months, and >12 months). P values were obtained using mixed models in which time into the study was included as a continuous variable and subject as a random factor for intercept. Mean values were obtained from all individuals and error bars indicate standard errors (between individuals).

**Figure 5:**
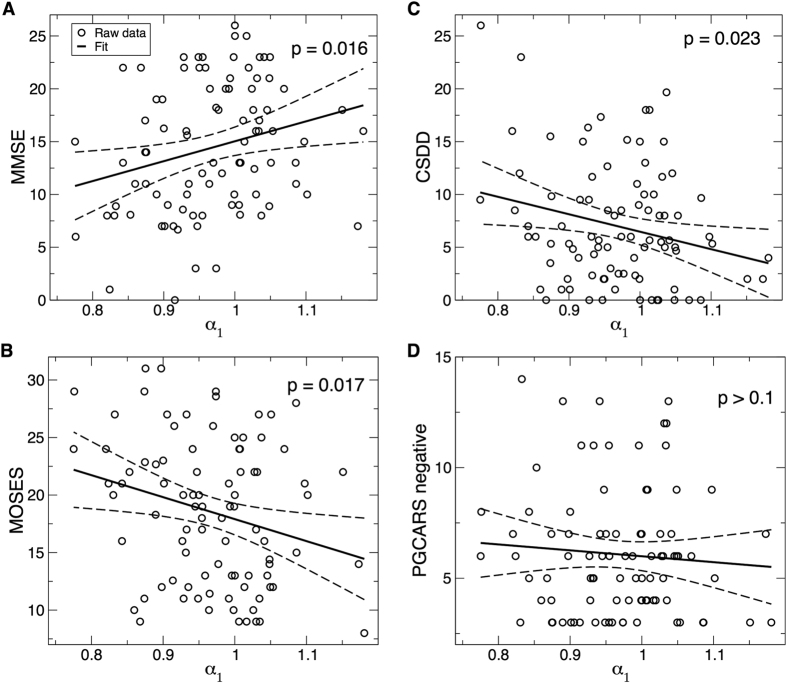
Association between functional outcomes and activity correlations at small time scales at baseline. (**A**) Mini-mental state examination (MMSE). (**B**) A subscale of the multidimensional observation scale for elderly subjects (MOSES) for withdrawn behavior. (**C**) Cornell Scale for Depression in Dementia (CSDD). (**D**) Negative affect scale of the Philadelphia Geriatric Centre Affect Rating Scale (PGCARS negative). Solid lines represent the predicted mean values and dashed lines are the 95% confidence intervals.

Similar to α_1_, cognition, depression, and psychological functions also showed significant changes from the baseline to the follow-up assessments, indicating functional deterioration over time (Fig. 4). At baseline, α_1_ was significantly associated with the Mini–Mental State Examination (MMSE) score (18.8 (7.7) × α_1_, r = 0.25, t ratio = 2.5, p = 0.016), the Cornell Scale for Depression in Dementia (CSDD) score (−16.5 (7.1) × α_1_, r = −0.24, t ratio = −2.31, p = 0.023), and the multidimensional observation scale for elderly subjects (MOSES) social withdrawal behavior score (−19.1 (7.8) × α_1_, r = −0.25, t ratio = −2.44, p = 0.017) (Fig. 5). These associations indicate that subjects with attenuated activity correlations at small time scales had worse cognition, mood, and social withdrawal behavior.

**Figure 6:**
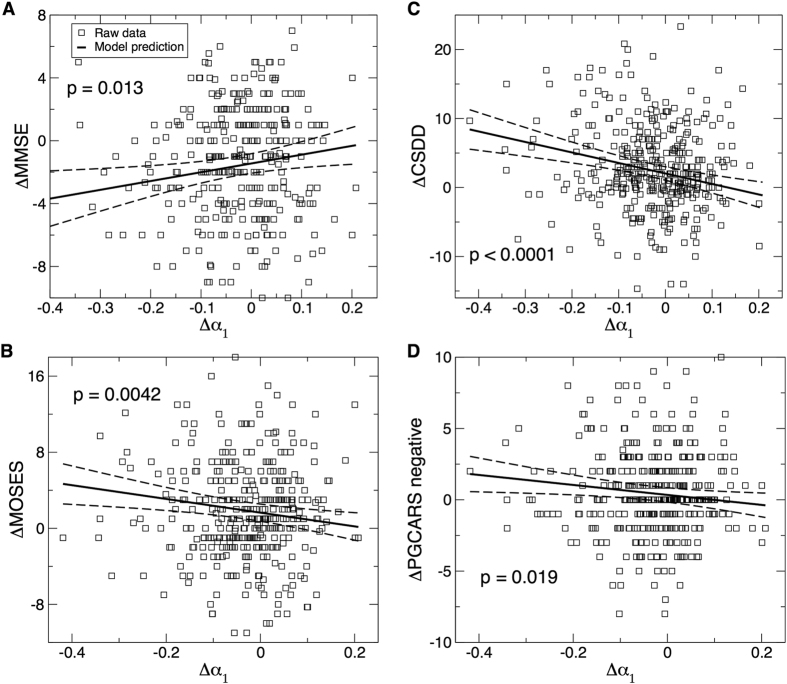
Association between the changes of functional outcomes and changes in activity correlations at small time scales from baseline to the follow-up assessments. (**A**) Mini-mental state examination (MMSE). (**B**) A subscale of the multidimensional observation scale for elderly subjects (MOSES) for withdrawn behavior. (**C**) Cornell Scale for Depression in Dementia (CSDD). (**D**) Negative affect scale of the Philadelphia Geriatric Centre Affect Rating Scale (PGCARS negative). P values were obtained from mixed models in which all raw data were included (i.e., not binned categorized variable) with subject as a random factor for the intercept. Solid lines represent the predicted mean values and dashed lines are the 95% confidence intervals.

During the follow-up assessments, mixed models showed that the change in α_1_ (Δα_1_: the change from the baseline or the first assessment) was: (i) positively correlated with the change of MMSE (ΔMMSE~5.6 (2.2) × Δα_1_, r = 0.11, t ratio = 2.49, p = 0.013); (ii) negatively correlated with the change of CSDD (ΔCSDD~−15.2 (3.5) × Δα_1_, r = −0.19, t ratio = −4.31, p < 0.0001); (iii) marginally associated with the increase in the Philadelphia Geriatric Centre Affect Rating Scale (PGCARS) negative affect scale (ΔPGCARS~−3.5 (1.5) × Δα_1_, r = −0.09, t ratio = −2.35, p = 0.019); and (iv) negatively correlated with the changes of withdrawn behavior scale of MOSES (ΔMOSES~−7.2 (2.5) × Δα_1_, r = −0.10, t ratio = −2.88, p = 0.0042) (Fig. 6). These associations indicate that less reduction in short time scale activity correlations over the years was associated with less cognitive decline, less mood decline and less increase in social withdrawal, respectively. We did not find any significant associations between any changes in activity correlation >2 hours (Δα_2_) and the changes of clinical outcome measures (all p values > 0.1).

## Discussion

Human activity fluctuations possess a complex, multiscale temporal organization which is significantly altered in older individuals and under pathological conditions (e.g., dementia and mood disorders)^5^,^7^,^8^,^9^,^10^,^11^,^12^,^13^. The current study provides the first analysis of within-subject changes of the temporal organization over time, the relevance of such changes to functional declines, and the effect of clinical interventions in people with dementia. Our results show that the temporal activity correlations at <~2 hours gradually decline over the years in patients with dementia and that this decline is associated with declines in mood and cognitive function. More importantly, we discovered that bright light treatment attenuated the progressive reduction in activity correlations by ~73%, suggesting for the first time that it is possible to manipulate human multiscale activity regulation with an intervention. The beneficial effect of the light treatment on multiscale activity regulation is consistent with its effects on cognition and depression symptoms. Using the same database, we previously reported that the light treatment attenuated cognitive deterioration by 5% on the MMSE and depression symptoms by 19% on the CSDD^31^. Since it is believed that the positive health impacts of bright light treatment are via its influences on SCN functioning and sleep-wake cycles, our findings support the notion that circadian regulation plays an important role in the temporal organization of activity fluctuations at multiple time scales^15^. Together these findings provide evidence that temporal activity regulation may be clinically relevant.

Frequent assessments of cognitive function and behavior are required to monitor improvement, stabilization or worsening of the disease-related symptoms in dementia patients and to better follow the progression of the disease. Regular assessments are also encouraged even in older persons without previously diagnosed dementia so that appropriate actions can be taken to prevent or delay the onset of dementia^32^. Our results suggest that the dynamic measure of activity fluctuations may serve as a promising tool for a routine assessment of cognition and behavior in older individuals. As compared to traditional clinical methods, this approach has a number of advantages in terms of cost efficiency and flexibility. For instance, the measure can enable a continuous assessment when subjects undergo their normal daily activities without the necessity of scheduling a visit with a doctor, physician, or other health care professional. With technical advance in data transmission and storage, collected activity data and results can be reviewed instantaneously or retrospectively to identify dynamic changes in patients’ conditions from day to day and even at different times of day. In addition, the tested activity measure (i.e., temporal correlations) is resilient to external influences of scheduled behavior and environmental conditions (e.g., daily schedule of interactions between dementia patient and caregivers in a nursing home)^6^. For instance, we have found that alterations in temporal activity correlations can better reflect intrinsic SCN neurodegeneration in dementia when compared to traditional circadian measures such as the amplitude of core body temperature (CBT) rhythm, the amplitude of motor activity rhythm, and intraday variability in motor activity — a measure characterizing fragmentation of the activity rhythm^10^. Thus, the application of this nonlinear measure to the clinic may potentially simplify monitoring of dementia progression and provide valuable data for a better physiological understanding of the disease and its impact on daily life.

How activity correlations at small time scales (<~1–2 hours) are altered in mood disorders is still unclear. Three recent studies provided seemingly discrepant results^4^,^12^,^13^. The study of Krane-Gartiser *et al*. reported more random activity fluctuations in acutely admitted inpatients with bipolar disorder^12^. Though based on different analytical tools to examine activity fluctuation (i.e., autocorrelation with lag = 1), the finding is consistent with our finding that the reduction of activity correlations at <2 hours was associated with more depression symptoms and more impaired cognition in patients/individuals with dementia. However, the two other studies suggested that mood disorders lead to increased temporal correlations in activity fluctuations at small time scales. Aybey *et al*. showed an increased DFA exponent in patients with major depression^4^ and Sano *et al*. reported persistence of resting and active periods in Schizophrenia^13^. The diversity of the deviations from normal is reminiscent of how correlations in heartbeat fluctuations are altered differently under different pathological conditions, i.e., heartbeat correlations at >~30 beats are reduced in arterial fibrillation but are increased in congestive heart failure^15^,^33^. It is possible that varied disorders or different stages of a disease may have differing influences on temporal activity correlations. Thus, further studies are required to clarify the changes of the activity correlations at small time scales for specific phases of specific diseases.

A similar concern applies to activity correlations at larger time scales. We previously found that activity correlations at time scales >~2 hours are reduced with aging and in dementia and that the reduction is related to the perturbed circadian regulation^9^,^10^,^30^,^34^. Since light and melatonin treatments are believed to be beneficial for circadian regulation in dementia patients, it would be expected that these treatments may also improve activity correlations at large time scales, but we did not observe such an effect in the current study. We note that most of our participants in this study were in the middle to late stages of AD/dementia (e.g., 88.5% of participants had MMSE ≤20 and only 19 participants had MMSE >20) and that the baseline activity correlations of the participants at large time scales were already significantly reduced (Figs 1 and 2) when compared to healthy young subjects and were similar to those of late-stage AD patients previously reported (α_1_ = 0.94 ± 0.03; α_2_ = 0.69 ± 0.03; both p values > 0.1 [t-test])^9^,^30^. Thus, perhaps activity correlations at large time scales already had maximally degraded during earlier stages of dementia and AD due to dysfunction of the circadian pacemaker^15^. Alternatively, perhaps the follow-up duration was too short (only 83 individuals remained in the study after 12 months). We favor the first hypothesis because, while activity correlations at small time scales progressively decreased over the follow-up periods, those at large time scales remained stable regardless of treatment. To definitively distinguish these two possible mechanisms, studies of older people at the early or preclinical stage of the diseases as well as a longer follow-up period are warranted.

As a first pilot investigation of within-subject changes in multiscale activity regulation, there are many notable limitations in the current study. One major potential concern is regarding the observed association between changes of activity correlations and cognition (MMSE). Since depression impairs both cognition^35^ and motor activity^5^,^11^,^12^,^13^,^14^, is it possible that the association was a simple indirect influence of worsening of depression with progression of dementia in our study? We have performed two additional statistical analyses to examine (1) the effect of changes in activity correlations on MMSE changes after controlling the effect of changes in Cornell Scale for Depression in Dementia (CSDD); and (2) the effect of changes in activity correlations on CSDD after controlling the effect of changes in MMSE, respectively. We found that the association between changes in activity correlations and MMSE became not significant (p > 0.1) while the association between changes in activity correlations and CSDD became less but still significant (r = −0.13; t ratio = −2.91, p = 0.0039; before accounting for the effect of MMSE: r = −0.19, t ratio = −4.31, p < 0.0001). These results suggest that the observed association between degraded activity correlations and cognitive decline might be due to the influences of depression on cognition and temporal activity regulation. However, due to the high correlation between the changes of MMSE and CSDD over time (r = −0.80, t ratio = −5.67; p < 0.0001) in the studied sample, the datasets are not ideal to tease out the complex causal links between depression, cognition, and multiscale activity regulation. In addition to depression, other neuropsychiatric symptoms such as hallucinations/delusions are also prevalent in dementia^36^. How these different symptoms contribute to perturbed temporal activity patterns was not examined in the current study and is worth further investigation. Moreover, many other pathological factors (different from cognition and mood) such as physical disabilities, cardiac disorders, and metabolic diseases may affect mobility and, thus, can have independent influences on activity correlations. We could not examine the potential role of these factors in degradation of activity regulation and its association with cognition in this study because no in-depth formal health examination/evaluation was made during the assessments that occurred every six months.

The other concern is that temporal activity correlations could only account for low percentages of variations in cognition and depression symptoms in patients/individuals with dementia, i.e., weak associations. Data integrity and study design might contribute to the weak associations. For instance, the activity recordings and the clinical measures were not collected simultaneously; and cognition and mood may vary significantly from time to time (e.g., time of day, and time of year) while these outcome measures were not obtained at the same time for each assessment during the longitudinal study. Though activity correlations appear to be independent of the mean activity levels in healthy young individuals (e.g., correlations remain the same at home at different days and in the laboratory with restricted daily schedule and physical activity)^6^, no studies have formally tested the effects of daily schedule and home environments in older adults, especially in dementia patients whose daily activities rely much on their caregivers. In addition, multiscale activity regulation may also be temporarily altered by certain conditions such as shift work via their influences on circadian control^34^. In this study, we calculated the correlations for each subject during each assessment using the whole recording while ignoring the potential variations in the correlations within the period of 1–2 weeks. Thus, the combination of non-simultaneous assessments and variations in activity control and clinical outcomes and might contribute to their weak associations.

Furthermore, the current study is focused on patients with dementia. Many neurodegenerative disorders can contribute to dementia such as AD, frontotemporal disorders, and Lewy body dementia. The progression of dementia and its impacts on behavior may differ for different types of dementia. The current study reveals the longitudinal changes of temporal structure in daily activity patterns in a heterogeneous population without distinguishing specific types of dementia. Future studies are warranted to examine whether within-subject changes of temporal activity correlations, their associations with cognition and mood, and their response to light are different in different types of dementia. In addition, within-subject changes of activity regulation in older persons without dementia are yet to be determined.

## Methods

### Subjects

To test our hypotheses, we analyzed activity recordings collected from an existing database of 189 residents^31^. These residents (170 women and 19 men; 70–96 years old; mean [SD] age: 85.7 [5.6] years) were living in assisted care facilities located in 12 different Dutch homes for the older individuals. The lowest age was data-driven rather than based on any cut-off exclusion criterion. These residents had their own apartment where they slept and retreated, but spent most of the daytime in a common living room supervised by caregivers. Among the 189 residents, there were 121 with probable AD based on the NINCDS-ADRDA criteria, 20 with probable vascular dementia and 24 with probable other types of dementia, based on the Diagnostic and Statistical Manual of Mental Disorders (Fourth Edition) criteria for dementia and dementia subtypes^37^, 17 without dementia, and 7 without sufficient information for reliable clinical diagnosis. The baseline demographic and clinical characteristics of subjects have been previously published^31^.

In a 2 × 2 factorial design, the 12 homes for the older individuals were randomly assigned to active (6 facilities, n = 98) or placebo (6 facilities, n = 91) light exposure, and subjects to double-blind daily intake of bedtime melatonin (n = 95) or placebo (n = 94). Thus, there are four groups: *Group 1*: 49 subjects assigned to active light and a placebo tablet; *Group 2*: 46 to inactive light and melatonin; *Group 3*: 49 to both active light and melatonin, and *Group 4*: 45 to inactive light and a placebo tablet. In this double blind randomized controlled trial, the four groups had no significant differences in age, sex, use of medication at inclusion and at any follow-up, and vision complications such as lens opacity and glaucoma^31^. The goal of this study is to determine the changes in temporal activity regulation and the clinical relevance in dementia patients. Thus, we studied all patients with confirmed dementia in the database (n = 165; 70–96 years old), including 45 patients in Group 1, 39 in Group 2, took bedtime melatonin each day, 44 in Group 3, and 37 in Group 4. These patients were diagnosed with dementia primarily, not with Major Depressive Disorder although some were treated because of mood symptoms. The 4 groups did not differ with respect to the proportion of participants receiving psychotropic medication including antidepressants, antipsychotics, anxiolytics and hypnotics at the onset of their participation (χ^2^-tests, all p > 0.32). Logistic mixed-effect regression analysis showed that the use of prescription drugs did not change after treatment onset compared with prescription use prior to treatment onset (all p > 0.80). There were no effects on prescription with either light or melatonin treatment or their interaction (all p > 0.35).

Informed consent was obtained from all subjects’ responsible relatives. The Medical Ethics Committees of Hospital De Gelderse Vallei, Ede, and the VU University Medical Center, Amsterdam, the Netherlands, approved the study. The methods were carried out in accordance with the approved guidelines.

### Study procedure

Participants were followed for up to 3.5 years, with a mean (±SD) of 15 (±12) months. Recruitment and enrollment commenced in 1999 and data acquisition continued until 2004. Follow-up assessments were made 6 weeks after the start of the treatment, and subsequently every 6 months. For each assessment, activity recordings and functional outcomes were assessed as described below.

Melatonin (2.5 mg, Terafarm, Brielle, the Netherlands) was given to *Groups 2* and *3* approximately 1 hour before bedtime by the nursing staff who ensured adherence. The tablets took about 1 hour to completely dissolve in water (a medium-fast release preparation). Timing and dosage were based on previous studies^38^,^39^. Placebo was given to *Groups 1* and *4* according to the same schedule as for the other two groups.

Light exposure was manipulated by installing a large number of ceiling-mounted fixtures with Plexiglas diffusers containing an equal amount of Philips TLD 840 and 940 fluorescent tubes (Philips Lighting BV, Eindhoven, the Netherlands) in the common living room of each of the selected 6 facilities. Illumination levels were obtained at intervals throughout one day at each assessment using a lux meter held at eye level in the direction of gaze, which was usually slightly downward or at best representing light falling on the vertical plane. A total of 3017 assessments were made. Average light exposure measured at eye level in the gaze direction was increased to ~1000 lux between 10 AM and 6 PM at the facilities randomized to the active light condition (P < 0.01 for all hourly comparisons between 10 AM and 6 PM of the active condition versus baseline except between 3 and 4 PM). The intensity for the active light treatment condition is enough to synchronize circadian rhythms in healthy people in a time-free environment^40^ and to improve daily/circadian activity rhythm disturbances in older patients with dementia^41^. For the placebo group in the other 6 facilities, an equal number of fixtures with only half of the tubes and concealed band-stop filters were installed at a greater distance from the eyes to achieve an exposure of ~300 lux. This clinical trial was registered in controlled-trials.com on December 9, 2005 (isrctnIdentifier:ISRCTN93133646). Clinical outcomes from this trial of bright light and melatonin in dementia have previously been published^31^.

### Data acquisition

For each assessment that was performed at baseline, after 6 weeks of treatment onset, and subsequently every 6 months after treatment onsets, motor activity levels were continuously monitored for 1–2 weeks using an Actiwatch (Cambridge Neurotechnology, Cambridge, England) worn on the wrist of the non-dominant hand. Acceleration was sampled at 32 Hz and was integrated to a proprietary ‘count’ value every minute.

The primary clinical outcomes are cognitive function assessed with the mini–mental state examination (MMSE)^42^, and mood with the Cornell Scale for Depression in Dementia (CSDD)^43^,^44^. In addition, we considered two secondary outcome measures: the Philadelphia Geriatric Centre Affect Rating Scale (PGCARS)^45^ and the withdrawn behavior subscale of the multidimensional observation scale for elderly subjects (MOSES)^46^.

### Activity correlations at multiple time scales

To assess activity regulation at multiple time scales, we performed detrended fluctuation analysis (DFA) to examine temporal correlations in the activity fluctuations at time scales from ~0.1 up to 12 hours. This method quantifies the detrended fluctuation function, F(n), of activity fluctuations at different time scales n (Fig. 1)^47^. To eliminate the effect of possible linear trends in original data, we applied the 2^nd^ order DFA, i.e., the 2^nd^ order of polynomial functions were used to detrend data when calculating F(n)^48^. A power-law form of F(n) indicates self-similarity (scale-invariance) in the fluctuations, yielding F(n)~n^α^. The parameter α, called the scaling exponent, quantifies the correlation property in the signal as follows: if α = 0.5, there are no correlations in the fluctuations (“white noise”); if α > 0.5, there are positive correlations, where large activity values are more likely to be followed by large activity values (and vice versa for small activity values). The exponent α = 1.0 indicates highest complexity in the systems^33^,^49^. Similar α values close to 1.0 have been observed in many physiological outputs under normal conditions, indicating a biological system with complex temporal regulations^33^.

In this study, we focused on multiscale activity regulation at time scales ≤12 hours (instead of up to 24 hours) during daytime. We made the decision for a number of reasons. (1) Scheduled 24-hour events, as likely occurred in assisted care facilities, can significantly affect activity fluctuations at time scales close to 24 hours (i.e., a masking effect)^9^. As previously reported, activity correlation and its changes with aging and in dementia can be properly identified using only daytime data^6^,^9^. (2) During the scheduled sleep episodes, activity of participants was somehow ‘restricted’ because they stayed in their own apartment without access to other facilities. To avoid the potential effects of imposed daily scheduled events, data during the individually assessed scheduled bedtimes (nurse informants) were excluded for analysis. Consequently, the derived activity measures reflect activity regulation that is more independent of sleep dynamics. Previous cross-sectional studies suggested that scale-invariant correlations are disrupted with aging and in dementia, leading to distinct correlations over two time scale regions with the boundary at ~1.5–2 hours^9^,^10^. Thus, for each activity recording in this study, we calculated the scaling exponent in two regions, separately, i.e. α_1_ at <90 minutes and α_2_ at >2 hours, omitting the variable transitional region of time scales between 1.5–2 hours.

Activity recordings of 21 dementia patients were either missing or were too short to be used for the correlation analysis. Thus, DFA results of 144 dementia subjects were reported in this study, including 40 (29 probable AD) in Group 1, 34 (25 probable AD) in Group 2, 39 (29 probable AD) in Group 3, and 30 (18 probable AD) in Group 4.

### Statistics

Follow-up time, temporal correlations of activity fluctuations, and measures of cognitive performance and mood were evaluated as continuous variables. Statistical analyses were performed using JMP Pro 11 (SAS Institute, Cary, NC). ANOVAs were used to determine the group differences in temporal correlations of activity fluctuations and measures of cognitive performance and mood at baseline. Mixed models with subject as a random factor for intercept were used to determine the effects of follow-up time, treatment with light, melatonin and their interaction on temporal correlations of activity fluctuations. Mixed models subsequently assessed the associations of changes in temporal correlations of activity fluctuations with changes in the measures of cognitive performance and mood that occurred from baseline to the final follow-up assessment.

## Additional Information

**How to cite this article**: Hu, K. *et al*. Progression of Dementia Assessed by Temporal Correlations of Physical Activity: Results From a 3.5-Year, Longitudinal Randomized Controlled Trial. *Sci. Rep.* **6**, 27742; doi: 10.1038/srep27742 (2016).
